# Granulomatosis with polyangiitis presenting with intestinal obstruction: A case report

**DOI:** 10.1016/j.ijscr.2022.107446

**Published:** 2022-07-21

**Authors:** Reem Alawna, Tala Jalamneh, Maram Massad, Noor Alawna, Abdalaziz Rabaia, Fadi Abu Alrub

**Affiliations:** a Jenin Governmental Hospital, Jenin, West Bank, Palestine; b Saad Dahlab University, Blida, Algeria

**Keywords:** GPA, granulomatosis with polyangiitis, Granulomatosis with polyangiitis, Intestinal obstruction, Wegener granulomatosis, Case report

## Abstract

**Introduction and importance:**

Granulomatosis with polyangiitis is a rare vasculitis. The gastrointestinal symptoms and complications of the disease are rare in GPA patients. One the rarest is intestinal obstruction which when found was always caused by bowel perforation.

**Case presentation:**

Here we report the case of a 14 years-old female patient who is a known case of granulomatosis with polyangiitis presented with intestinal obstruction without perforation and treated conservatively.

**Discussion:**

Gastrointestinal involvement is seen in only 5–10 % of the cases and is a poor prognostic factor. This life-threatening complication could be caused by bowel perforation and as we describe here with bowel inflammation only without perforation.

**Conclusion:**

Intestinal obstruction without perforations is a new complication of GPA. And conservative management should be considered in the plan of treatment.

## Highlights

• Gastrointestinal involvement is a poor prognostic factor.
• Active inflammatory process located in the gastrointestinal tract in the course of GPA is a rare complication; however, causing a severe threat to the lives of patients.
• Case of GPA can present with intestinal obstruction without perforation and it can be treated conservatively.

## 1 Introduction

Granulomatosis with polyangiitis (GPA) - previously known as Wegener granulomatosis - is a medium and small-vessel vasculitis which affects the respiratory tract and the kidneys. Necrotizing granulomatous inflammation characterizes the clinical condition [1] with the presence of anti-neutrophil cytoplasmic antibodies (cANCA). The most common affected site causing clinical manifestations in GPA is the upper airway [2], less commonly; the gastrointestinal system manifestations include oral mucosa ulcerations, gum mucosa hypertrophy, dyspepsia, vomiting, stomachache, gastrointestinal hemorrhage, diarrhea, and symptoms of gastrointestinal tract perforation [3]. But none of the cases reported in the literature presented solely with bowel obstruction without perforation.

## 2 Case presentation

**Fig. 1:**
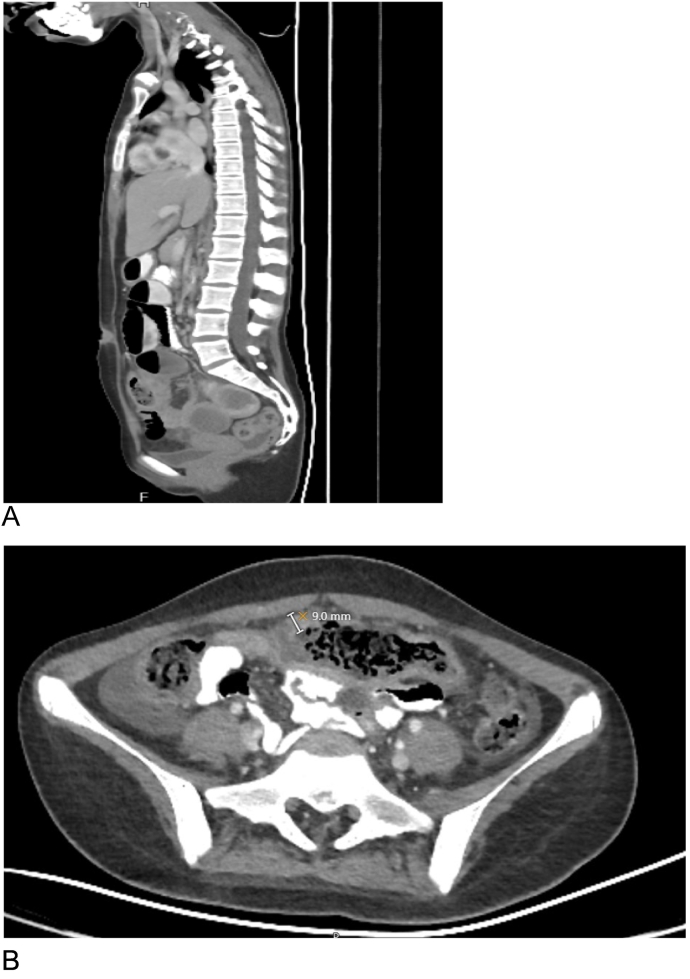
CT scan; sagittal view with oral contrast: shows multiple air-fluid levels within visualized small bowel loops. B: Axial CT scan with IV and oral contrast shows; increased transverse bowel wall thickness of around 1 cm.

**Fig. 2:**
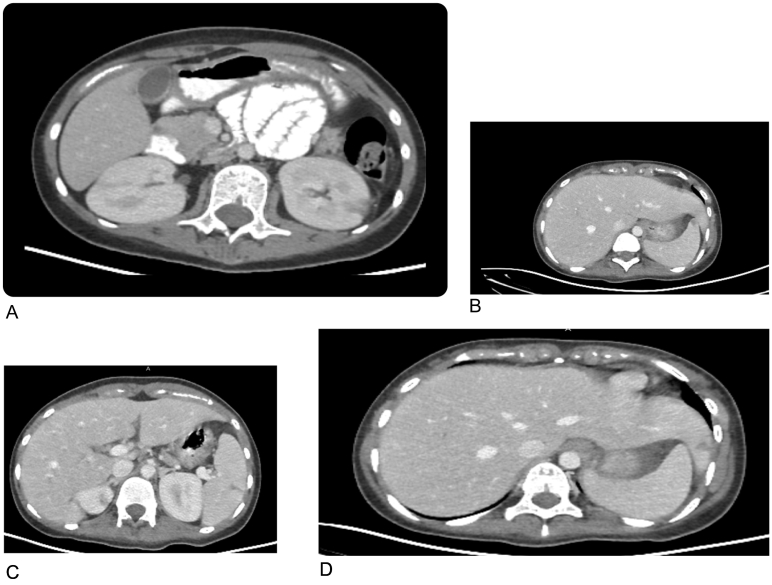
A: Axial CT scan with IV and oral contrast portal-venous phase: shows; wedge shaped hypodense areas in both kidneys, the largest on the left side measuring about 1.3 × 0.8 cm representing bilateral renal infarcted areas. B: Axial CT scan with IV contrast in portal venous phase: Two small hypodense areas are seen within the spleen measuring 0.4 ∗ 1 cm. C: Axial CT scan with IV contrast in portal venous phase: Two small hypodense areas are seen within the spleen measuring 0.7 ∗ 1 cm. D: Axial CT scan with IV and oral contrast portal venous phase: small hypodense area triangular shape seen in the left liver lobe measuring about 1 ∗ 0.6 cm presenting a focal small infarcted area.

A 14 year-old female patient who is a known case of GPA presented to the emergency department complaining of colicky abdominal pain. She was doing well until 2 weeks before the admission when she started to complain of abdominal pain, which increased in intensity in the last 10 h before admission, the pain had no radiation, partially relieved by analgesics and associated with multiple episodes of non-bloody vomiting, decreased appetite and constipation of one-day duration. No history of mouth ulcers, dysphagia, odynophagia, melena or hematochezia. No respiratory symptoms were reported. The patient medical record showed a history of pulmonary aspergillosis. On exam, the patient was moderately dehydrated, in pain, and afebrile. The abdomen was distended, tympanic with increased bowel sounds and mildly tender all over. CT with and without oral and IV contrast for abdomen and pelvis showed evidence of bowel obstruction (Fig. 1A, B) and multiple lesions in the liver spleen and kidneys consistent with infarctions (Fig. 2A, B, C, D).

The patient was diagnosed with intestinal obstruction, and no physical or imaging signs of perforation were found. The case was managed conservatively with a nasogastric tube for decompression. She was kept on methylprednisolone sodium succinate, appropriate antiemetic, analgesia, a PPI and the standard hydration. After 3 days she showed improvement and was discharged a day later after the resolution of symptoms.

## 3 Methods

We reported one case of GPA presented with intestinal obstruction without perforation, we reviewed English-language literature regarding gastrointestinal manifestations of GPA especially intestinal obstruction.

We report our case structure according to the SCARE Guidelines 2020 and checklist [8].

## 4 Discussion

GPA is a medium and small-vessel vasculitis that causes necrotizing granulomatous inflammation [1]. The upper respiratory tract is involved in 70–100 % of cases of GPA [4]. In the head and the neck, the disease mainly involves the nasal cavity and the paranasal sinuses and less commonly the ear [5] which may lead to permanent facial nerve paralysis, permanent sensorineural [6], conductive, and mixed hearing loss [7]. The most common anatomical site for lesions in GPA is the upper airway. GPA can also affect the eyes, skin, joints and nervous system [2] and the kidneys. The gastrointestinal system may be affected as well. The main symptoms related to the gastrointestinal tract were: oral mucosa ulcerations, gum mucosa hypertrophy, dyspepsia, vomiting, stomachache, gastrointestinal hemorrhage, diarrhea, and symptoms of gastrointestinal tract perforation [3].

GI manifestations are seen more often in males, active inflammatory process located in the gastrointestinal tract in the course of GPA is a rare complication; however, it does occur, causing a severe threat to the lives of patients. To this date, at least 11 cases have been reported in the literature [1]. Masiak A et al. also concluded in their study that GI manifestation can be the presenting symptom of GPA and predicts the involvement of other organ systems [3]. Intestinal perforation affects intestinal function and peristalsis causes signs and symptoms of bowel obstruction and this was always the case for GPA patients until we discovered this case. After thorough revision of the literature and to the best of our knowledge, this is the first case of GPA presenting with bowel obstruction without perforation. This reported case expands the horizon of differential diagnosis of GPA cases presenting with abdominal pain. And adds the conservative management as an option for management of intestinal obstruction in GPA patients.

## 5 Conclusion

GPA patients can present with intestinal obstruction without perforation and this complication can be treated conservatively.

## Consent

Written informed consent was obtained from the patient for publication of this case report and accompanying images.

## Provenance and peer review

Not commissioned, externally peer-reviewed.

## Ethical approval

Not applicable - no need.

## Funding

This research received no specific grant from any funding agency in the public, commercial, or not-for-profit sectors.

## Guarantor

Dr. Abd Al-aziz Rabaya.

## Research registration number

1. Name of the registry: not available
2. Unique identifying number or registration ID: not available
3. Hyperlink to your specific registration (must be publicly accessible and will be checked): not available.

## CRediT authorship contribution statement

All authors contributed to conception of the case, gathering patient's information, manuscript writing and reviewing.

## Declaration of competing interest

The authors have no conflict of interest to declare.
